# A state of delirium: Deciphering the effect of inflammation on tau pathology in Alzheimer's disease

**DOI:** 10.1016/j.exger.2016.12.006

**Published:** 2017-08

**Authors:** Matthew Barron, Jane Gartlon, Lee A. Dawson, Peter J. Atkinson, Marie-Christine Pardon

**Affiliations:** a School of Life sciences, University of Nottingham, Queens Medical Centre, Nottingham NG7 2UH, UK; b Eisai Inc., 4 Corporate Drive, Andover, MA 01810, USA; c Astex Pharmaceuticals, 436 Cambridge Science Park Rd, Cambridge CB4 0QA, UK; d Eisai Ltd., EMEA Knowledge Centre, Mosquito Way, Hatfield, Hertfordshire, AL10 9SN, UK

**Keywords:** Alzheimer's disease, Tau, Phosphorylation, Inflammation, Preclinical models, Lipopolysaccharide

## Abstract

Alzheimer's disease (AD), the predominant form of dementia, is highly correlated with the abnormal hyperphosphorylation and aggregation of tau. Immune responses are key drivers of AD and how they contribute to tau pathology in human disease remains largely unknown. This review summarises current knowledge on the association between inflammatory processes and tau pathology. While, preclinical evidence suggests that inflammation can indeed induce tau hyperphosphorylation at both pre- and post-tangles epitopes, a better understanding of whether this develops into advanced pathological features such as neurofibrillary tangles is needed. Microglial cells, the immune phagocytes in the central nervous system, appear to play a key role in regulating tau pathology, but the underlying mechanisms are not fully understood. Their activation can be detrimental *via* the secretion of pro-inflammatory mediators, particularly interleukin-1β, but also potentially beneficial through phagocytosis of extracellular toxic tau oligomers. Nevertheless, anti-inflammatory treatments in animal models were found protective, but whether or not they affect microglial phagocytosis of tau species is unknown. However, one major challenge to our understanding of the role of inflammation in the progression of tau pathology is the preclinical models used to address this question. They mostly rely on the use of septic doses of lipopolysaccharide that do not reflect the inflammatory conditions experienced AD patients, questioning whether the impact of inflammation on tau pathology in these models is dose-dependent and relevant to the human disease. The use of more translational models of inflammation corroborated with verification in clinical investigations are necessary to progress our understanding of the interplay between inflammation and tau pathology.

## Highlights

• Inflammation modulates tau function in Alzheimer's disease.
• LPS induces tau phosphorylation *in vivo*.
• Modulation of late stage tau pathology is less clear.
• Microglial shows potential to slow spread of extracellular tau.
• A holistic approach will determine the role of inflammation in Alzheimer's disease.

## 1 Introduction

**Fig. 1:**
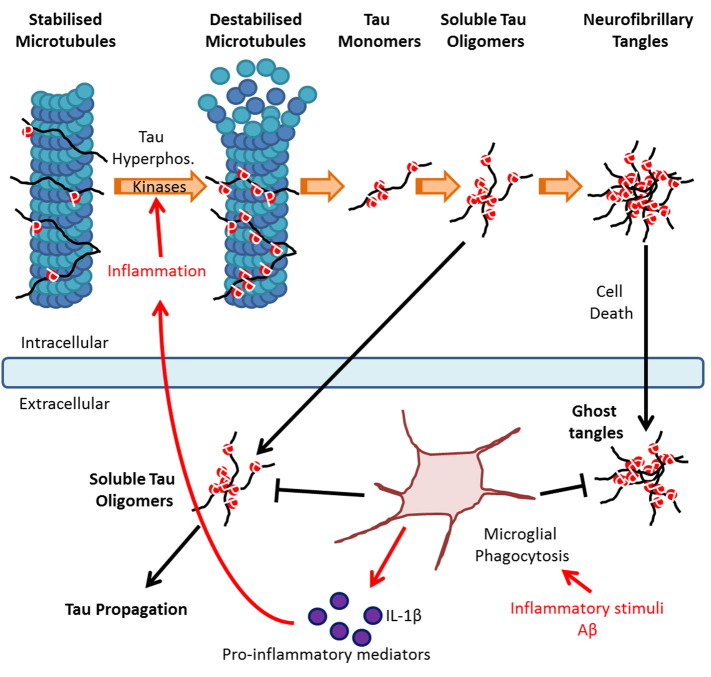
Progression of tau pathology: Under physiological conditions tau regulates microtubule stabilisation. In tauopathies, tau hyperphosphorylation triggers a loss in microtubule affinity. Soluble tau aggregates into pathological soluble tau oligomers, ultimately forming pathological insoluble neurofibrillary tangles (NFT). Tau oligomers are secreted into the extracellular compartment contributing to the propagation of tau pathology into neighbouring neurons. Inflammatory stimuli, such as Aβ, stimulate microglial production of pro-inflammatory mediators such as IL-1β leading to the up-regulation of kinases involved in tau phosphorylation and exacerbation of the pathology. However, inflammation can have beneficial effects on tau pathology by inducing microglial phagocytosis of extracellular tau species. Image adapted from National Institute of Ageing.

Alzheimer's disease (AD) is classified under a group of neurodegenerative diseases termed tauopathies owing to its association with tau pathology. Tau is a microtubule-associated protein predominately expressed in neurons, which stabilizes microtubules under physiological conditions, and as such regulates axonal stability and cell morphology (Avila et al., 2004). Under pathological conditions such as AD, tau is abnormally hyperphosphorylated leading to a decrease in its affinity for microtubules, a process represented in Fig. 1. Soluble hyperphosphorylated tau then aggregates into pathological soluble and insoluble aggregates known as neurofibrillary tangles (NFT), a hallmark of AD. In addition to NFT, amyloid-beta (Aβ) plaques are identified in AD brains, however cognitive decline correlates to a greater extent with tau pathology (Schöll et al., 2016).

Inflammation is considered a key mechanistic driver in AD where both Aβ plaques and NFT co-localize with microglia and astrocytes, the resident immune cells of the brain (Serrano-Pozo et al., 2011). Genome wide association studies (GWAS) suggest a strong association between AD and genes involved in the regulation of immunological function (Lambert et al., 2013), whereas epidemiological studies have revealed a reduced risk of developing the disease following long-term anti-inflammatory treatments (Vlad et al., 2008) and disease-exacerbating effects of infectious agents (Honjo et al., 2009). Corroborating these observations, pro-inflammatory stimuli have been shown to induce both amyloid and tau pathologies in animal models (Zilka et al., 2012).

To date, several randomised trials of anti-inflammatory agents have been conducted in AD. Despite the use of multiple types anti-inflammatory treatments, whether non-steroidal anti-inflammatory drugs (NSAID) or steroidal, all have failed to demonstrate clear clinical efficacy in AD patients (Jaturapatporn et al., 2012). Pathogenesis in AD develops years prior to symptom manifestation, and therefore, anti-inflammatory agents were suggested to be beneficial when administered prodromal. Although the largest trials assessing NSAIDs on subjects at risk of developing AD have failed to show benefits on AD incidence (Breitner et al., 2013) a recent updated systematic review still argues in favor of their use for prevention of AD (Wang et al., 2015).

The mechanisms underlying the involvement of immune responses in AD pathogenesis remain poorly understood because inflammation has both beneficial and detrimental effects which can be very context dependent (Heneka et al. 2016). Aβ pathology is exacerbated through the induction of pro-inflammatory mediators secreted from active immune cells such as microglia (Brugg et al., 1995) but conversely, activation of these cells can stimulate clearance of Aβ plaques *via* induction of phagocytosis (Fiala et al., 2007), demonstrating a dual role for inflammation on amyloid pathology. Less is known about the specific role of inflammatory processes on tau pathology, and to our knowledge, the effect of anti-inflammatory treatments on tau pathogenesis in humans is unknown. In preclinical models inflammation is generally seen as an exacerbating factor (Zilka et al., 2012) but recent data suggest that it may be beneficial as well (Majerova et al., 2014). Here, we will review the preclinical findings to shed light on the interplay between inflammation and tau pathology in AD.

## 2 Inflammation induces tau phosphorylation

Table 1 summarises the outcomes of studies assessing the effect of pro-inflammatory stimuli on tau pathology.

**Table 1:** Studies reporting the effect of inflammation on tau pathology.

Model	Challenge	Time to cull	Effect on tau	Kinases implicated	Reference
Primary neuronal and microglia cultures	LPS (30 ng/ml)	n/a	Tau phosphorylation (epitope not specified)	↑ P38 MAPK	Li et al. (2003)
Primary neuronal and microglia cultures	IL-1β (30 ng/ml)	n/a	Tau phosphorylation (epitope not specified)	–	Li et al. (2003)
3xTg-AD (Amyloid + Tau)	LPS (6 weeks, twice per week, 0.5 mg/kg, *i.p.*)	24 h	↑ pT231/pS235,	↑ CDK5,	Kitazawa et al. (2005)
			↑** pS202/pT205,**	= GSK-3β,	
			=** pS396/404**	= JNK,	
				= p38 MAPK	
3xTg-AD (Amyloid + Tau)	LPS (6 weeks, twice per week, 0.5 mg/kg, *i.p.*)	48 h	↓ Total tau,	↑ GSK-3β	Sy et al. (2011)
			↑** pS202/pT205,**	= CDK5	
			↑** pS214,**		
			↑** pT212/pS214,**		
			=** pT212**		
			↑ pS396/404 containing insoluble tau		
3xTg-AD (Amyloid + Tau)	MHV (*i.p.*)	2 weeks/4 weeks	↑** pS396/404**	↑ GSK-3β,	Sy et al. (2011)
				↑ CDK5	
rTg4510 (Tau)	LPS (10 μg, *i.c.v*)	1 weeks	↑** pS199/pS202,**	–	Lee et al. (2010)
			↑** pS396**,		
			= Insoluble tau		
hTau (Tau)	LPS (1 mg/kg, *i.p.*)	24 h	↑** pS202/pT205,**	–	Bhaskar et al. (2010)
			↑ pT231		
C57BL/6 (WT)	LPS (10 mg/kg, *i.p.*)	24 h	↑** pS202/pT205,**	–	Bhaskar et al. (2010)
			↑ pT231,		
			↑** pS396/404**		
C57BL/6 (WT)	LPS (100 μg/kg, *i.p.*)	0–4 h	Transient: ↑** pS396/404,**	↑ GSK-3β,	Roe et al. (2011)
				↑ CDK5,	
				= ERK2,	
				= JNK	
			↑** pS202/pT205**		
3xTg-AD (Amyloid + Tau)	R-flurbiprofen (10 mg/kg, daily, 2 m, chow)	–	= pS202	–	Carreras et al. (2013)
			=** ps202/pT205**		
			↓** ps396/404**		
3xTg-AD (Amyloid + Tau)	Ibuprofen (daily, 5 months, chow)	–	↓** ps202/pT205**	–	McKee et al. (2008)
hTau (Tau)	Minocycline (10 mg/kg daily, 14d, *i.p.*)	2 h	↓ ps202,	–	Noble et al. (2009)
			↓** pS396/404**		
			↓ Insoluble tau		

Bold indicates tau phosphorylation epitopes associated with post-tangle pathology (Augustinack et al., 2002).

### 2.1 Systemic immune stimuli induce neuroinflammation

The majority of our understanding for the role played by inflammation on tau pathology relies on the use of systemic immune challenges, and particularly of the toll-like receptor 4 (TLR-4) agonist; lipopolysaccharide (LPS) which fails to cross the blood brain barrier (Banks and Robinson, 2010), thereby mimicking systemic infections. LPS nevertheless induces central inflammatory responses through a variety of including neural routes such as vagal afferents, humoral routes through circumventricular organs, infiltration of peripheral monocytes and through effects on brain endothelial cells (Miller and Raison, 2016, Pardon, 2015), and as such can affect tau pathology.

### 2.2 Inflammation induces tau phosphorylation in tau models

The first direct evidence for a role of inflammation in exacerbating tau pathology stemmed from *in vitro* studies with primary microglial cells stimulated with Aβ or LPS prior to being co-cultured with primary neocortical neurons (Li et al., 2003). This landmark study showed that secretion of the pro-inflammatory cytokine interleukin-1β (IL-1β) by microglial stimulation causes an increase in tau phosphorylation through activation of p38-mitogen-activated protein kinases (MAPK). This has been confirmed *in vivo* predominantly using the 3xTg model which exhibits both tau and amyloid pathologies (Oddo et al., 2003). A chronic treatment regimen with LPS (0.5 mg/kg twice a week for 6 weeks) triggered tau hyperphosphorylation at multiple phosphorylation sites associated with both pre- and post-tangle tau pathology in 3xTg mice, and at both early and advanced pathological stages (Kitazawa et al., 2005, Sy et al., 2011). Again, microglial activation and resulting secretion of IL-1β were implicated, *via* activation of either cyclin dependent kinase-5 (CDK-5) (Kitazawa et al., 2005) or glycogen synthase kinase-3β (GSK-3β) (Sy et al., 2011). The discrepancy in the kinases involved is likely due to differences in age and pathological stages between the two studies. Infection with an altered murine hepatitis virus (MHV) strain likewise triggered an increase in tau phosphorylation demonstrating the ability of both bacterial and viral immune stressors to induce tau phosphorylation (Sy et al., 2011). Chronic overexpression of another pro-inflammatory cytokine, tumor necrosis factor-α (TNFα), also caused an increase in the pre-tangle-associated pT231 epitope (Janelsins et al., 2008). But in contrast, when the pro-inflammatory cytokine interferon-γ (IFNγ), which is predominately associated with viral infections, was overexpressed, tau dephosphorylation occurred at pre-tangle associated phosphorylation sites (Mastrangelo et al., 2009). Altogether, this suggests that whether or not inflammation triggers exacerbation of tau pathology is, at least in part, dependent on the nature of immunological stressor and the pro-inflammatory cytokine involved. However, the fact that chronic treatment with the NSAIDS: ibuprofen or r-flurbiprofen were found to induce tau dephosphorylation at post-tangle associated phosphorylation epitopes (Carreras et al., 2013, McKee et al., 2008), despite their poor brain penetration (Parepally et al., 2006), suggests that basal peripheral inflammation contributes to the development tau pathology.

While the 3xTg model is useful for studying interactions between amyloid and tau pathologies, tau specific models are valuable to decipher whether inflammation directly affects tau pathology. One such model is the rTG4510 mouse which expresses a repressible form of human tau containing the P301L mutation that has been linked with familial frontotemporal dementia (SantaCruz et al., 2005). An intra-hippocampal injection of 10 μg of LPS in these mice resulted in an increase in tau phosphorylation persisting for at least seven days post injection (Lee et al., 2010), confirming that neuroinflammation can directly modulate tau phosphorylation independently of amyloid pathology. Models in which a mutated form of tau is expressed exhibit a more severe pathology than those based on non-mutated tau, although they are of lower relevance to AD. The hTau model expresses all 6 non-mutated tau isoforms as in AD, leading to pathological tau aggregation (Andorfer et al., 2003). Both pre- and post-tangle phosphorylation sites were shown to be increased following a 1 mg/kg dose of LPS in the hTau model, demonstrating that the disease-exacerbating effect of inflammation is not caused by tau mutations *per se* (Bhaskar et al., 2010). To address the mechanisms involved, the authors used hTau mice deficient for the fractalkine receptors which are exclusively expressed on microglial cells in the central nervous system and known to stimulate anti-inflammatory and pro-phagocytic responses (Medina and Ravichandran, 2016). This intervention exacerbated the impact of LPS on tau phosphorylation (Bhaskar et al., 2010), again supporting a role for microglia function in pathological tau phosphorylation, but also suggesting that activation of the protective immunosuppressive and phagocytic phenotype of microglia could be beneficial to tau pathology.

### 2.3 LPS induces tau phosphorylation in wild-type (WT) mice

While transgenic models provide relevance to pathogenesis of human tau, the use of wild-type (WT) mice has proved useful to examine LPS effects on non-pathogenic tau. Interestingly, a low 100 μg/kg dose of LPS was found to induce CDK5-dependent tau phosphorylation at post-tangle associated epitopes as early as 20 min post-injection, and which subsided within 4 h (Roe et al., 2011) indicating that low levels of inflammation are sufficient to transiently trigger tau phosphorylation. Conversely, Bhaskar et al., 2010 demonstrated that a septic dose of LPS (10 mg/kg) was not associated with changes in tau phosphorylation 24 h following administration in WT mice. The failure to observe tau hyperphosphorylation could be attributed to the use of a later time-point than Roe et al. (2011). However, when the mice were deficient of the fractalkine receptor, the same 10 mg/kg dose, an increase in microglial activation and tau phosphorylation was seen 24 h later at both pre- and post-tangle sites, and IL-1 receptor signalling was again found to be the underlying mechanism (Bhaskar et al., 2010). Taken together, these findings demonstrate that pro-inflammatory stimuli induce tau hyperphosphorylation at epitopes associated with both pre- and post-tangle pathology in both WT and transgenic models of AD.

## 3 What constitutes an alteration in tau pathogenesis?

### 3.1 Are changes in tau phosphorylation sufficient to draw conclusions on tau pathology?

**Fig. 2:**
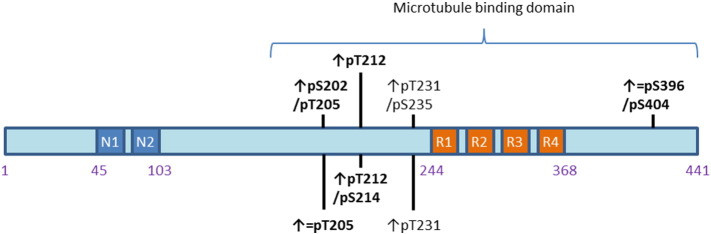
Tau phosphorylation sites which have been shown to be affected by inflammatory stimuli: Bold sites indicate post-tangle compared to pre-tangle associated phosphorylation sites. ↑ indicates increase and = indicates no change. ↑ = indicates an increase or unaltered depending on the study. Picture adapted from (Barré and Eliezer, 2013) and phosphorylation states from (Augustinack et al., 2002).

Tau phosphorylation represents a mechanistic trigger for a pathological cascade which ultimately leads to the development of NFT. Both pre- and post-tangle associated phosphorylation epitopes were found to be induced following immune stimulation as summarized in Fig. 2, therefore the question arises as to the relative importance of specific phosphorylation epitopes. Indeed the association between tau phosphorylation and development of pathology is poorly understood, with increases in tau phosphorylation not always developing into tau aggregation. For instance, Lee et al. (2010) showed in the rTG510 model that while tau phosphorylation was increased following acute central LPS administration, tau aggregation remained unaffected. This indicates that assessing tau phosphorylation is not necessarily representative of an impact on tau pathology. In contrast, Sy et al. (2011) reported an increase in tau aggregation following a chronic LPS treatment regimen in the 3xTG model. Interestingly the same phosphorylation epitopes were affected in the two studies (Lee et al., 2010, Sy et al., 2011) possibly demonstrating a role of acute *vs.* chronic effects, although a role for amyloid in the latter study cannot be ruled out. In support of this hypothesis, a chronic reduction in basal inflammation induced through the tetracycline antibiotic: minocycline reduced levels of both phosphorylated and aggregated tau in the hTau model (Noble et al., 2009). Recent advancements have suggested tau oligomers as the tau species with the greatest pathogenic potential, with their accumulation at the synapses, rather than NFT, being thought to cause synaptic dysfunction (Guerrero-Muñoz et al., 2015). Tau oligomers have the unique ability to translocate across the synapse, propagating tau pathology into healthy neighbouring neurons in a process known as tau seeding (Guerrero-Muñoz et al., 2015). Assessing the effect of inflammation on extracellular tau species will enable greater insight into our understanding of the effects of inflammation on tau pathology.

### 3.2 Do inflammatory processes have a protective role in the development of tau pathology?

While the literature to date points towards a detrimental role of inflammation in exacerbating tau pathology, recent evidence suggests a novel beneficial role. As observed with amyloid pathology, this is attributed to microglial phagocytosis of tau species. Indeed, Majerova et al. found that both primary and immortalised microglial cells stimulated with LPS can phagocyte synthetic extracellular tau oligomers (Majerova et al., 2014). While this demonstrates that microglia have the propensity to phagocytose tau oligomers, caution is required as the concentration of the oligomers used was much greater than physiological *in vivo* concentrations (Majerova et al., 2014). C57BL/6 mice injected with both soluble and aggregated human tau likewise show microglial internalization of both species of tau (Bolos et al., 2015). Since the soluble tau injected included both monomeric and oligomeric tau, this provides indirect evidence that microglia have the potential to phagocytose toxic tau oligomers. Inflammation could therefore be providing a beneficial effect through inhibiting the spread of tau pathology by microglial phagocytosis of extracellular tau seeds as described in Fig. 1.

## 4 Is the inflammatory response modelled relevant to AD?

The data discussed above points towards dose-dependency in the effects of inflammation on tau pathology, questioning the relevance of the stimuli used to model AD pathogenesis. LPS doses higher than 1 mg/kg are considered to simulate sepsis rather than infection (Thomas et al., 2014), a condition less frequently observed in AD patients. Only one study used a dose below this threshold, 100 μg/kg, which is thought to reflect mild systemic infection (Murray et al., 2012) and only resulted in transient increases in tau phosphorylation (Roe et al., 2011). While chronic LPS treatment regimens, as used by Kitazawa et al. (2005), might be more relevant to AD pathogenesis, tolerance occurs with repeated LPS injections (Pardon, 2015), and this has to be taken into account when trying to identify which inflammatory processes contribute to the tau pathology. Furthermore, LPS models only bacterial infections, which is not representative of the range of inflammatory conditions experienced by AD patients.

### 4.1 Concluding remarks

Inflammation has been suggested to play a role in the development of tau pathology, but the underlying mechanisms remain poorly understood. While IL-1β signalling in microglia appears to be detrimental, the data discussed here questions the pertinence of using tau phosphorylation as a readout for tau pathology. Pro-inflammatory stimuli robustly induces tau phosphorylation in model systems, but whether or not this progresses into the pathological process of tau aggregation is largely unknown. Novel evidence also suggests a beneficial role for inflammation on tau pathology through induction of microglial phagocytosis of tau oligomers, with the potential to inhibit the spread of tau pathology. Thus, suppressing the immune system could prove paradoxical at pathological stages of AD by potentiating tau pathological seeding into healthy neurons (Heneka et al., 2016). Finally, the doses at which LPS were found to induce tau phosphorylation in mice are more representative of sepsis in humans, indicating that a substantial inflammatory response is needed to induce tau pathology in pre-clinical models. Through modelling mild and chronic infection akin to that seen in AD, we will be able to better understand the role played by inflammation in pathogenesis and treatment of AD.

## Conflict of interests

Jane Gartlon and Peter Atkinson are employees of Eisai Ltd., and Eisai Inc., Lee Dawson is an employee of Astex Pharmaceuticals.
